# Improving serious illness communication: a qualitative study of clinical culture

**DOI:** 10.1186/s12904-023-01229-x

**Published:** 2023-07-22

**Authors:** Joanna Paladino, Justin J. Sanders, Erik K. Fromme, Susan Block, Juliet C. Jacobsen, Vicki A. Jackson, Christine S. Ritchie, Suzanne Mitchell

**Affiliations:** 1 Massachusetts General Hospital, Boston, MA USA; 2 Harvard Medical School, Boston, MA USA; 3 Ariadne Labs, Joint Innovation Center at Brigham & Women’s Hospital and Harvard T.H. Chan School of Public Health, Boston, MA USA; 4 Mongan Institute Center for Aging and Serious Illness, Division of Palliative Care and Geriatric Medicine, Massachusetts General Hospital, Boston, USA; 5 McGill University, Montreal, CA Canada; 6 Dana Farber Cancer Institute, Boston, MA USA; 7 Lund University, Lund, Sweden; 8 UMass Memorial Health Care, Worcester, MA USA

**Keywords:** Serious illness communication, Goals of care, Patient-provider communication, Quality improvement, Clinical culture

## Abstract

**Objective:**

Communication about patients’ values, goals, and prognosis in serious illness (serious illness communication) is a cornerstone of person-centered care yet difficult to implement in practice. As part of Serious Illness Care Program implementation in five health systems, we studied the clinical culture-related factors that supported or impeded improvement in serious illness conversations.

**Methods:**

Qualitative analysis of semi-structured interviews of clinical leaders, implementation teams, and frontline champions.

**Results:**

We completed 30 interviews across palliative care, oncology, primary care, and hospital medicine. Participants identified four culture-related domains that influenced serious illness communication improvement: (1) clinical paradigms; (2) interprofessional empowerment; (3) perceived conversation impact; (4) practice norms. Changes in clinicians’ beliefs, attitudes, and behaviors in these domains supported values and goals conversations, including: shifting paradigms about serious illness communication from ‘end-of-life planning’ to ‘knowing and honoring what matters most to patients;’ improvements in psychological safety that empowered advanced practice clinicians, nurses and social workers to take expanded roles; experiencing benefits of earlier values and goals conversations; shifting from avoidant norms to integration norms in which earlier serious illness discussions became part of routine processes. Culture-related inhibitors included: beliefs that conversations are about dying or withdrawing care; attitudes that serious illness communication is the physician’s job; discomfort managing emotions; lack of reliable processes.

**Conclusions:**

Aspects of clinical culture, such as paradigms about serious illness communication and inter-professional empowerment, are linked to successful adoption of serious illness communication. Further research is warranted to identify effective strategies to enhance clinical culture and drive clinician practice change.

**Supplementary Information:**

The online version contains supplementary material available at 10.1186/s12904-023-01229-x.

## Introduction

A cornerstone of person-centered care, particularly in the context of serious illness, is high-quality communication with patients about their prognosis, values, and goals (‘serious illness communication’) [1]. People living with serious illness and their caregivers experience high burdens of suffering and are at risk of receiving care that is not aligned with their priorities [1]. Yet fewer than one-third of individuals with serious illness report having a conversation about their goals with their clinician [2]. When conversations do occur, they often take place near the end of life and neglect psychosocial, emotional, and cultural needs [3]. These gaps contribute to poor quality care and avoidable suffering for patients and caregivers and moral distress for clinicians [1, 4].

Researchers and practitioners have created and spread many interventions to improve serious illness communication. One such intervention, the Serious Illness Care Program (SICP), is a multifaceted intervention that includes structured communication tools (Serious Illness Conversation Guide, or Guide), clinician communication skills training, and system-changes, including processes to identify patients, reminders in the workflow, and an electronic health record (EHR) documentation template [5–7]. Research on SICP demonstrates improvements in serious illness conversations, lower rates of anxiety and depression for patients, and positive patient experiences [8–14]. Clinicians also described significant improvements in communication skills [15] and meaningful experiences, including enhanced satisfaction in their role [9, 16].

While effective in research settings, serious illness conversations can be challenging to implement in routine care. Prior SICP implementation research [17] revealed the importance of organizational context and implementation strategies in facilitating adoption of serious illness conversations by clinicians. However, researchers have yet to examine the contextual domain of clinical ‘culture,’ or the attitudes, beliefs, and norms of health professionals in relation to serious illness communication [18, 19]. Little is known about the specific aspects of culture that influence successful implementation of serious illness conversations in real-world quality improvement initiatives. In this qualitative analysis, we studied the experiences of health professional implementers from five systems that adopted SICP to characterize the aspects of clinical culture that supported or impeded improvements in serious illness communication.

## Methods

### Settings

This study included five health systems in Massachusetts, Pennsylvania, Texas, and California that are implementing SICP as educational and quality improvement initiatives. All five settings are academic medical centers with integrated healthcare delivery systems (ranging from ~ 600 to ~ 1,700 beds). SICP implementation occurred across specialties, including (for example) oncology, primary care, geriatrics, palliative care, and hospital medicine. These systems were chosen as a purposive convenience sample based on: (1) implementation for 3 years or more; (2) commitment to system-wide improvement; (3) use of SICP resources.

### Design and data collection

We conducted a qualitative study using semi-structured interviews of health professional participants involved in implementing the program. The methodology and reporting conform to the Standards for Reporting Qualitative Research [20]. The study was approved by the Mass General Brigham Institutional Review Board as exempt. Interviews were conducted between February 2020 and April 2021. The principal investigator (JP), a female palliative care researcher and faculty with SICP, recruited participants and conducted interviews. Recruitment occurred through individual e-mail solicitations and snowball sampling. Invited participants were health professionals with diverse professional and clinical roles related to SICP, including: a) teams who oversee institutional SICP implementation (implementation teams); b) frontline clinicians involved with improvement efforts (frontline champions); c) specialty leaders involved in SICP processes (clinical leaders). The sampling strategy and size was informed by the principle of ‘information power,’ which proposes that sufficient information power depends on: the aim of the study, sample specificity, use of established theory, quality of dialogue, and analysis strategy [21]. Our aim was narrow; the participants were deeply knowledgeable and experienced in the topic area (SICP and serious illness communication); we utilized an established conceptual framework (see below); the interviewer had expertise in the content area and the quality of the interviews was high. JP conducted qualitative interviews until data saturation was achieved, the point at which the interviews produced redundant and no new information [21, 22]. Participants received a $25 Amazon gift card.

### Interview framework

A semi-structured interview guide was used across all interviews (Supplemental Table 1). Following informed consent, interviews lasted 60 min and were audio-recorded, transcribed, and anonymized. Interview guide development was based on prior SICP implementation research informed by the Promoting Action on Research Implementation in Health Services (PARIHS) implementation framework [17, 18, 23] and a conceptual framework on patient safety culture that includes organizational, unit/interpersonal, and individual domains [24–26]. Interviews focused on: successes and challenges of improving serious illness conversations; individual, interpersonal, implementation, and organizational factors that influenced improvement.

### Data analysis

We employed a stepwise approach to thematic analysis [27, 28]. The primary analytic team was comprised of two researchers with extensive qualitative research experience (JP; SM, female family medicine physician and health services researcher, not involved with SICP implementation). JP and SM reviewed the first five transcripts and used open coding to develop an initial codebook, resolving differences by consensus. JP and SM coded the remaining transcripts and met every five interviews to revise and add codes and ensure iterative content building. To produce a finalized codebook, the codes emerging from this process were consolidated and organized deductively using the PARIHS framework [18, 23], dividing the coded data into organizational contextual factors (e.g. leadership, EHR, measurement), implementation factors (e.g. champion facilitation), and culture-related interpersonal and individual factors (e.g. knowledge, attitudes, beliefs). The final codebook was applied to all interviews. Rigor was maintained through regular conversations with a third researcher (JS male palliative care physician researcher) to link the codes into themes and sub-themes, looking for expansion or contradictions of evolving constructs. Preliminary findings were presented to a subset of participants (*n* = 5 implementation team leaders) for member checking, which confirmed the authenticity of the findings. Researchers discussed how personal clinical and implementation experiences inform and potentially influence interpretation of the data as a process to mitigate potential bias. Culture-related individual and interpersonal factors are reported here; organizational and implementation factors are reported separately.

## Results

### Participant characteristics

A total of 30 participants completed the interviews out of 35 invited (86% participation rate). Inter-professional participants were comprised of implementation teams (47%), frontline champions (37%), and clinical leaders (16%) (Table 1).

**Table 1:** Participant characteristics (*n* = 30)

	**Characteristics**	**n (%)**
**Program Role**	**Implementation Team**	**14 (47%)**
	MD	8
	PA	1
	Project or program manager	5
	**Frontline Champions**	**11 (37%)**
	MD	5
	NP/PA	2
	RN	2
	SW	2
	**Clinical Leaders**^**a**^	**5 (16%)**
	MD	5
**Specialty**^**a**^	Palliative care	9 (30%)
	Oncology	5 (17%)
	Hospital/ED/Surgery	5 (17%)
	Primary Care/Geriatrics	3 (10%)
	Dual-boarded^b^	3 (10%)
	Administrative	5 (17%)
**Gender**	Female	22 (73%)
	Male	8 (27%)
**Years in practice**	< 10 years	13 (43%)
	> = 10 years	17 (57%)
**Health System**	System 1	5
	System 2	6
	System 3	6
	System 4	7
	System 5	6

^a^Each system had participants that represented clinical leaders

^b^Three clinician participants are dual-boarded (2 in palliative care and oncology; 1 in palliative care and geriatrics)

### Qualitative results

SICP implementation surfaced domains of clinical culture that appeared to support or inhibit improvements in serious illness communication, including: (1) Clinical paradigms; (2) Interprofessional empowerment; (3) Perception of conversation impact; (4) Practice norms (Table 2, Additional/Supplemental Table 2).

**Table 2:** Supportive and inhibitive aspects of clinical culture of serious illness communication

Domain	Inhibitive culture aspects	Supportive culture aspects
**Clinical paradigms** • Knowledge & assumptions • Beliefs • Attitudes	• Association of serious illness communication with discussions about dying, hospice, or life-sustaining treatment decisions • Beliefs that serious illness conversations have a pre-ordained outcome of withdrawing or limiting curative care • Conflicted attitudes and discomfort toward communication	• Adoption of a definition of serious illness communication as knowing and honoring what matters to patients • Beliefs that the purpose of serious illness communication is to strengthen therapeutic relationships, provide emotional support, and enable partnership in all treatment decisions • Positive attitudes toward and comfort with eliciting patients’ values and goals
**Interprofessional empowerment** • Confidence and self-efficacy • Psychological safety and trust • Role identity	• Reluctance and nervousness about conversations with patients because of uncertainty about what to say • Perceptions that serious illness communication is the physician’s job; lack of psychological safety for inter-professional clinicians • Feeling uncertain about role and scope of practice in serious illness communication	• Improved comfort and self-efficacy in initiating serious illness conversations with patients • Acceptance of inter-professional roles in serious illness communication; enhanced psychological safety and trust • Integration of serious illness communication into professional roles
**Perceived impact** • Impact on patients • Impact on clinicians	• Concerns about taking away hope and increasing anxiety and/or sadness for patients • Feeling overwhelmed by serious illness communication due to discomfort with emotions and overburdened environments	• Perception that earlier values and goals conversations lessen distress for patients • Feeling more effective in personalizing care, more meaning and fulfillment at work, and stronger relationships with patients
**Practice norms** • Timing of conversations • Focus and content of conversations • Reliability and accountability	• Reactive approach to communication that ‘avoids’ conversations until a crisis or poor prognosis at end of life • Predominantly medically oriented content of the conversations e.g. hospice, code status, life-sustaining treatment preferences • Lack of reliable team processes or unclear roles, ‘chaos’ and ‘kicking the can down the road’ effects	• Integrating earlier and longitudinal conversations into practice • Enhanced focus of conversations on patients’ values, goals, hopes, and worries (rather than treatments and procedures) • Shared responsibility and accountability that integrates communication into team processes and norms

#### Theme 1: clinical paradigms

Clinical paradigms, or mental models that clinicians hold, encompassed three sub-themes: assumptions about what serious illness communication entails; beliefs about the intended outcome of a conversation; and attitudes toward conversations. Clinicians outside of specialty palliative care often associated serious illness communication or goals of care with conversations about dying, life-sustaining treatments, or hospice. Some clinicians surfaced beliefs that the pre-ordained outcome of a serious illness conversation was to ‘withdraw’ or ‘limit’ curative care, which engendered worries that engaging patients in these discussions would interfere with providing advanced therapies. These beliefs and associations contributed to conflicted attitudes, discomfort with, and stigma toward serious illness conversations, which emerged as barriers to improvement.

*A big thing as part of our project was instead of calling them goals of care or family meetings, we called them patient-centered care conferences....so that way, residents and the nursing staff didn't feel like ...'we're going to start withdrawing care.’ System 3, oncology nurse, frontline champion*

*“We spent about a year planning for SICP...and were only a few weeks away from training the nurses when...one of the...surgeons said to me, ‘You can't ask these questions because these patients may need a transplant and I don't want you to talk them out of a transplant.’” System 4, palliative care nurse practitioner, implementation team*

Participants also noted paradigm shifts related to serious illness communication that were internalized by some clinicians, which revealed cultural changes that supported improvement. Clinicians adopted a definition of serious illness communication that was ‘decoupled’ from acute crises or end-of-life planning and instead focused on getting to know patients personally and understanding and honoring what matters to patients. Clinicians also described serious illness conversations as a way of strengthening the therapeutic relationship, supporting patients emotionally, and ensuring partnership in all treatment decisions. These shifts were perceived to be a change in norms by participants.

*“This has prompted us to have the conversations at an earlier point than just six months. By asking the questions this way, it reframed for me- you can’t say what the choices for treatment are if you don’t know what is important to the patient, or what a patient is afraid of, or what they value most.” System 4, oncology physician, frontline champion*

*“I think I’ve seen it come the farthest within our [primary care] nurse care management group...their deep understanding and recognition of what a serious illness conversation is... and what it’s not. I think we are still up against the broader organization. People conflate code status and MOLST as a serious illness conversation when we’re really talking more upstream.” System 2, primary care program manager, implementation team*

*“These conversations are not just about end-of-life, it's about meeting patients where they're at...and asking them, what do you understand about what's going on with you? What are you worried about? What are you hopeful for?...at the core...is getting to understand the patient...” System 3, oncology nurse, frontline champion*

#### Theme 2: interprofessional empowerment

Empowerment encompassed three sub-themes: comfort and self-efficacy; perceptions about roles; and psychological safety. Participants identified discomfort, in part due to uncertainty about what to say, as a key contributor to clinician reluctance to initiate serious illness conversations. In addition, a common perception that serious illness communication was the ‘physician’s job’ contributed to hesitation by advanced practice clinicians, nurses, and social workers to engage patients in discussions, which led to feelings of powerlessness and fears that serious illness communication was not within their scope of practice. Lastly, participants described instances in which physicians expressed negative attitudes toward inter-professional roles by actively blocking the involvement of nurses and social workers in conversations. This created a psychologically unsafe environment that interfered with team adoption of serious illness conversations.

*“We had educational sessions [with nurses]...A lot of the stuff that we got back from people was that they were afraid that this was working outside of their scope to start these conversations...” System 3, oncology nurse, frontline clinician*

*“There's a department...where a [leader] said, 'No social worker may ever... have a conversation about end-of-life with our patients...’ And I have rarely gone to that [site].” System 2, palliative care social worker, frontline champion*

Participants, however, described positive cultural changes in empowerment that supported improvement in serious illness conversations. First, participants noted that use of a structured Conversation Guide helped to improve clinicians’ comfort and self-efficacy by providing language and a framework for conversations. Second, changes to physicians’ attitudes toward more acceptance of interprofessional roles enabled nurses and social workers to feel safe to initiate conversations proactively, rather than waiting for permission from a physician team member. Some noted, however, that generating physician trust and acceptance took time, in part because of paradigms that associate serious illness communication with sharing poor prognosis or end-of-life transitions. Third, informants noted shifts in role identity in which clinicians, especially nurses and social workers, felt increasing ownership of and accountability for ensuring that patients’ values and goals were addressed.

*“We really weren’t that confident that we were going to be allowed to do this type of work. There was a lot of timidity in the work at the onset...Over time, it has become very integrated into the work we do. I don’t wait to be directed to have the conversations. I take proactive steps.” System 2, primary care nurse, frontline champion*

*“I felt safe but not at the beginning...It took time to build that trust...[and to] convince the hospitalists...what my goal was- as the person who is working to make sure that the patient’s treatment is aligned with their goals.” System 2, inpatient palliative care social worker, frontline champion*

#### Theme 3: perceived conversation impact

Perceptions of the impact of serious illness conversations (as either positive or negative) influenced improvement and fell into two sub-themes: impact on patients and impact on clinicians. Clinician concerns that earlier serious illness conversations would take away patient hope or increase patient anxiety and sadness were common barriers. In addition, difficulties managing patients’ emotions, and distress in coping with their own emotions, were contributors to clinician avoidance of and discomfort with these discussions. The emotional and logistical size of the task felt overwhelming in typical environments due to scarcity from short visit lengths, high patient volumes, staff shortages, and competing priorities. These factors contributed to a default in which conversations were avoided or deprioritized compared to other tasks.

*“I think we probably don’t acknowledge this enough...but the actual pain involved for the clinician. So, these are deep and meaningful conversations, and I think they do cause some sadness for patients...and I think oncology clinicians don’t want to introduce more pain...” System 4, palliative care and oncology physician, implementation team*

“*For all of us doctors who are afraid of emotions...it’s easy to tiptoe around it if we have 15 minutes and a lot of other things to talk about.” System 3, hospitalist physician, implementation team*

*“[A colleague] emailed us a couple days ago and said, “I’m so sorry...we’ve just been so busy...I want to do this. I know it’s important. I can’t even get through my clinic day.’”* System 5, palliative care physician, implementation team

When earlier values and goals discussions were integrated into practice, participants observed benefits which encouraged sustainability of conversations. Creating space for patients to surface their goals, values, hopes, and fears without pressure to make decisions was perceived to alleviate patient distress and to better prepare patients and families for changes in the future. In addition, clinicians reported feeling more effective in their role in personalizing care by integrating patients’ values and goals more explicitly into decision-making, which was perceived to strengthen relationships, enhance patient or family satisfaction with decisions, and increase clinician confidence that care aligned with patients’ expressed wishes. For some clinicians, integrating serious illness conversations into practice enhanced meaning at work, returned them to why they went into healthcare, and facilitated working at the top of their skillset.

*“It’s definitely rewarding to have these conversations because...it feels like, “Oh, this is what we should be doing as social workers.” It’s bringing us back to our roots that we’re not just here to hand out substance use resources.” System 2, inpatient social worker, frontline champion*

*“I think allowing that SICP conversation to happen allowed [the family] to tell a story to personalize [the patient], and it makes it a lot easier for them to guide decision-making and realizing that you can help him by potentially supporting him through this difficult time and keeping him comfortable.” System 1, hospitalist physician, clinical leader*

#### Theme 4: practice norms

Participants characterized serious illness conversation practice norms as a key domain of clinical culture which included three sub-themes: timing of conversations; content of the discussions; reliability of team processes. Typical communication was observed to be reactive in which conversations were often avoided until patients had an acute crisis and/or a poor prognosis near the end-of-life. In part because of the late timing, discussions tended to focus on acute decisions, such as code status or hospice. In addition, inadequate or unreliable team processes (sometimes described as ‘chaos’), and lack of clarity about team roles, resulted in a ‘kicking the can down the road’ effect in which serious illness conversations did not occur reliably for patients.

*“I would say that there was chaos where there was a lot of transitioning in and out of our hospitals...and...you felt like somebody needs to talk to this family...but there was a feeling like whose responsibility is that? Is it the primary care doctor’s?...You weren’t sure that this was part of what you should be doing.” System 2, primary care nurse, frontline champion*

*“...serious conversations were usually a reactive process versus proactive. Oftentimes people came into the hospital sick with the expectation of getting better, and when things didn't, there was a reactive process...about making critical decisions when patients are heading down the tubes quickly.” System 1, hospitalist physician, frontline champion*

Participants noted, however, that supportive cultural changes appeared to enable behavioral patterns to shift in ways that improved the person-centeredness of serious illness communication. Participants described a more proactive approach to communication in which conversations about values and goals start earlier in the illness course and were more likely to be revisited over time, enabling patients to have a voice in their care before becoming too sick. Participants noted a change in the content of the discussions to focus more on patients’ goals, values, hopes, and worries. Participants provided examples of consistent team workflows that reflected shared responsibility and accountability for serious illness communication.

*“Content of the discussions....moving from...being focused very much on life-sustaining treatment preferences and code status to more values-based discussions about goals, preferences, patient values...we see that in the documentation that we're collecting....you can see the goals changing.” System 4, palliative care physician, implementation team*

*“When you enroll your patients, you do ask your physicians the surprise question, which triggers that kind of thought process that, okay, we have work to do...we need to have that conversation...So it becomes kind of a team effort early on.” System 2, primary care nurse, frontline champion*

## Discussion

**Fig. 1:**
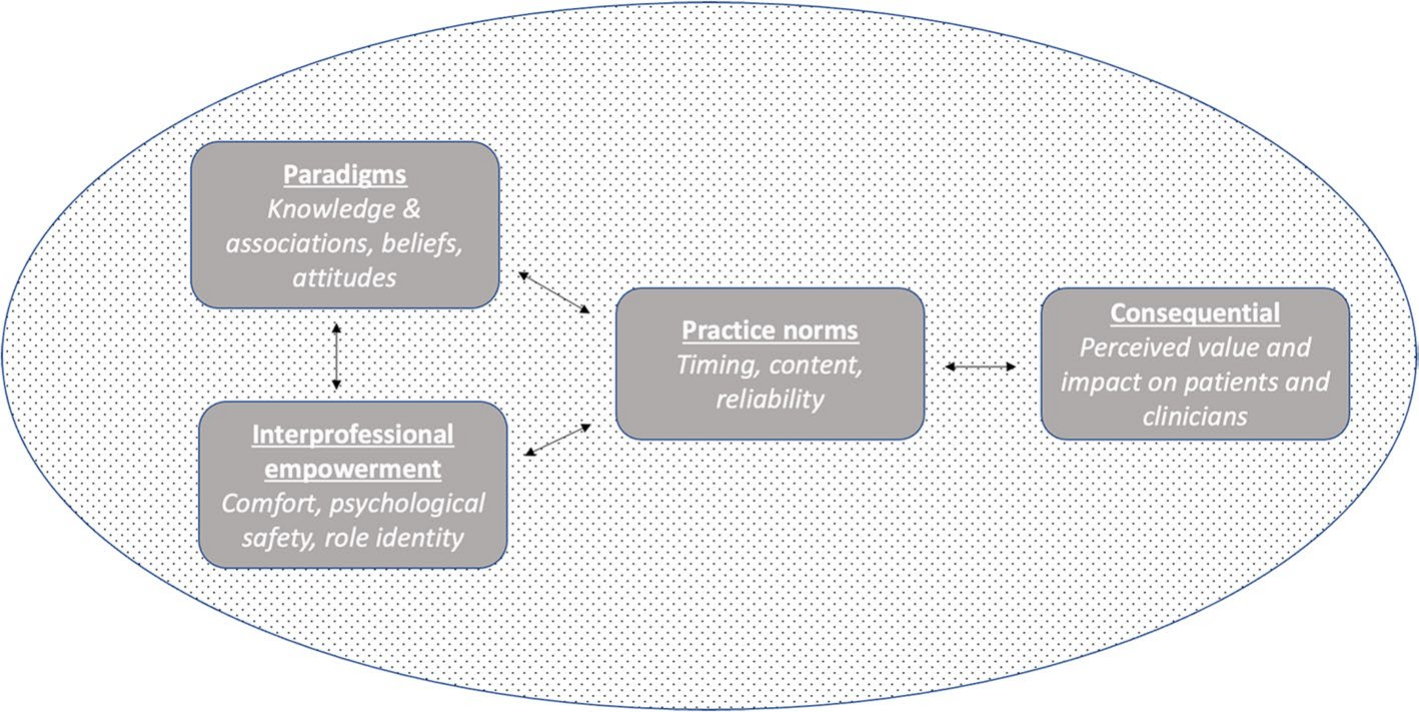
Domains of serious illness communication clinical culture

This qualitative study of Serious Illness Care Program implementation elucidated four inter-related domains of clinical culture (Fig. 1) that were perceived by implementers to support or inhibit improvements in serious illness conversations, including: (1) clinical paradigms; (2) interprofessional empowerment; (3) perceived conversation impact; (4) practice norms.

Implementation of the Serious Illness Care Program surfaced embedded attitudes and beliefs in our clinical culture that appeared to increase clinician discomfort with and stigma toward serious illness communication, which served as barriers to practice change. Clinicians across specialties outside of palliative care associated serious illness conversations with discussions about end-of-life planning or transitions, including life-sustaining treatment preferences, hospice care, or limiting curative care. Surfacing these narratives in the clinical culture may help identify underlying mechanisms of communication quality gaps. For example: prior research demonstrates a high prevalence of missed opportunities to engage patients with serious illness in values and goals conversations [29] as well as difficulties generating clinician practice change as part of quality improvement initiatives [17]. These findings also complement recent palliative care research which revealed public misperceptions about palliative care and related practices [30–32]. This study suggests, however, that a communication intervention can potentially shift clinician mindset and practices toward a more positive and person-centered view of serious illness communication, as a way of knowing and honoring what matters to patients, supporting patients emotionally, and strengthening therapeutic alliance and partnership in decisions [7, 33, 34], which builds on prior SICP research in the hospital setting [16]. Identifying specific culture-related narratives that may need to change raises important implications for implementation. For example: research on SICP as well as other quality improvement programs suggests that improvement efforts may benefit from culture change tactics, such as leadership strategies [17, 35, 36]; messaging and engagement to foster clinician self-reflection, connection, and learning [37–39]; and multi-level structure and process changes [17, 38, 40].

This analysis also revealed the importance of shifting from individual to team ownership of serious illness communication, which relied on interprofessional empowerment and psychological safety, respect, and acceptance of the role of advanced practice clinicians, nurses, and social workers in serious illness conversations, especially by physicians. However, attitudes that serious illness communication is the ‘physician’s job,’ ambiguity about roles [41], and unprofessional behaviors by physicians in some instances interfered with inter-professional clinicians feeling safe to engage patients in conversations. The association of serious illness communication with end-of-life transitions or sharing poor prognoses also added to the concerns that it was not within nursing and social work scope of practice. There is a substantial evidence base about the influence of psychological safety on quality and safety interventions [25, 42–44]. Future work could involve integrating team-based training models, such as simulation training for inter-professional clinicians on serious illness communciation [43, 45, 46], to enhance teamwork and collaboration, foster respect for diverse skillsets and roles, and support more members of the care team to feel safe, entrusted and empowered to engage patients in serious illness conversations. This finding also builds on prior research demonstrating the need to develop inter-professional training [47], resources [48], and models of serious illness communication delivery [49–52] to expand the workforce involved in these discussions, which may increase access for patients and families.

The findings in this study expand upon other research demonstrating positive clinician experiences with relationship-centered communication interventions [16, 53]. Enhancing the person-centeredness of serious illness conversations appeared to enable clinicians to feel more like their best selves, including strengthening connection with patients, providing more personalized care, and returning them to why they went into healthcare. However, this analysis revealed that conversations also had an emotional cost for clinicians. In an overwhelming climate in which clinicians are constantly being asked to do more with less [54], discomfort with surfacing and managing patients’ and their own emotions served as barriers to practice change. In addition to patient and caregiver experience and outcomes, future studies of serious illness communication interventions should incorporate attention to clinician well-being and experience [55]. Future research can also involve the development of a valid and reliable survey to assess clinician perceptions of serious illness communication culture, similar to patient safety culture surveys [24–26, 56, 57]. While culture has been identified as a barrier in serious illness care [58–60], culture in this area has not yet been defined. Making culture ‘visible’ through measurement may be a crucial facilitator of ongoing research, quality improvement, and program evaluation [61].

### Limitations

This is a qualitative analysis that relied on the subjective perceptions of health professionals at large academic health systems, which may not be generalizable to clinical culture in diverse settings. The background of the interviewer as a SICP palliative care faculty may have biased what participants shared. To mitigate this, the researcher used facilitative techniques to create safety for participants to share their authentic experiences. There were also limitations with the participant sample in this study. Participants included a smaller proportion of leaders than other groups. In addition, participants tended to be individuals who were proponents of serious illness conversations, including a sizable proportion of palliative care clinicians and program administrators who were on the implementation team and reported on their direct observations and experiences of working with colleagues in different specialties. This introduces a bias that limits the generalizability of the findings about clinical culture and the conclusions that can be drawn from this analysis. However, these perspectives are also an important part of studying culture, which encompasses witnessed interactions in addition to individual experiences [62]. In the next phase of research, it will be important to include a more diverse array of perspectives, including frontline clinicians who are not identified as champions; additional institutional leaders and administrators; and patients and caregivers.

## Conclusion

Changing clinician practice in serious illness communication appeared to rely on strengthening aspects of clinical culture that encouraged adoption of earlier values and goals conversations, such as: shifting clinical paradigms about serious illness communication; enhancing interprofessional empowerment; revealing benefits to patient care and clinician meaning; and generating shifts in practice norms through individual and team responsibility for earlier values and goals conversations. Identifying effective strategies to enhance clinical culture may improve the success of interventions in driving sustained improvements to person-centered serious illness communication practices.

## Supplementary Information

**Additional file 1.** Interview Guide.

**Additional file 2.** Themes and Sub-themes with Representative Quotes.

## Data Availability

All data relevant to this study are included in the article or uploaded as supplemental information.

## Abbreviations

SICP: Serious Illness Care Program
EHR: Electronic Health Record
