# Supplementary material for: Improving serious illness communication: a qualitative study of clinical culture
Source: BMC Palliat Care. 2023 Jul 22;22:104. doi: 10.1186/s12904-023-01229-x (PMC10362669; doi:10.1186/s12904-023-01229-x)
Supplement: Supplementary file 1 — Additional file 1. Interview Guide. [file 12904_2023_1229_MOESM1_ESM.pdf]

Additional File 1: Interview Guide

Interview Guide
1. What is your current role at your institution?<br>a. What is your role with the Serious Illness Care Program?<br>2. Why was the Serious Illness Care Program adopted to your knowledge?<br>a. Probe: How did serious illness conversations typically happen before implementation? What was the perceived impact of these typical SIC norms at baseline? Please provide specific examples and observations.<br>3. What are the successes of the initiative, if any? Please provide specific examples and observations.<br>a. Did serious illness conversations change over the course of implementation? If so, how did they change? Please provide specific examples and observations. If not, can you provide comments about why you think they haven't changed.<br>i. Probe: Have clinician perceptions about serious illness conversations changed over the course of the implementation of the Serious Illness Care Program?<br>1. If yes, can you describe how they have changed? If no, why do you think they haven't changed?<br>ii. Probe: What changes have occurred, if any, within clinical teams for serious illness conversations? If no changes, why do you think they haven't changed?<br>iii. Probe: What changes have occurred in the specialty or institution in terms of serious illness conversations? If no changes, why do you think they haven't changed?<br>b. What factors contributed to positive changes/successes?<br>i. Probe: Individual (e.g. attitudes, beliefs, skills, knowledge, practices)<br>ii. Probe: Interpersonal/team (e.g. team dynamics, roles, workflows)<br>iii. Probe: Implementation process (e.g. engagement, training, QI methods)<br>iv. Probe: Organizational and structural (e.g. leadership, resources, EHR, data, measures)<br>c. What factors interfered with positive changes/successes?<br>i. Probe: Individual (e.g. attitudes, beliefs, skills, practice norms)

    ii. Probe: Interpersonal/team (e.g. team dynamics, roles, workflows)
    iii. Probe: Implementation process (e.g. engagement, training, QI methods,)
    iv. Probe: Organizational and structural (e.g. leadership, resources, EHR, data, measures)

4. What are the major challenges you encountered with the initiative? Please provide specific examples and observations.
    a. What strategies did you use to try to overcome challenges? What worked? What didn't work? Please provide specific examples.
5. If a colleague was starting an initiative to improve serious illness communication, what recommendations would you give them based on your experience and lessons learned?
6. Is there anything else you would like to share that you haven't already?
