# Supplementary material for: Improving serious illness communication: a qualitative study of clinical culture
Source: BMC Palliat Care. 2023 Jul 22;22:104. doi: 10.1186/s12904-023-01229-x (PMC10362669; doi:10.1186/s12904-023-01229-x)
Supplement: Supplementary file 2 — Additional file 2. Themes and Sub-themes with Representative Quotes. [file 12904_2023_1229_MOESM2_ESM.pdf]

Additional File 2: Themes and Sub-themes with Representative Quotes

	**Supportive culture aspects**	**Inhibitive culture aspects**
Theme: Clinical paradigms	**Sub-themes:** Perceptions that serious illness communication is to know and honor what matters most to patients longitudinally (as opposed to end-of-life planning); beliefs that earlier values and goals conversations are an integral aspect of the therapeutic relationship; attitudes toward communication (e.g. patient's values and priorities should be integrated into longitudinal partnership in decision-making)<br><br>*These conversations are not just about end-of-life, it's about meeting patients where they're at and understanding what they know and what they understand and what they want. And whether that's somebody who has a terminal oncology diagnosis, or somebody that's in the hospital for a hip replacement, all you're doing is asking them, what do you understand about what's going on with you? What are you worried about? What are you hopeful for? And if that conversation then progresses to somebody who has terminal cancer talking about their prognosis, or talking about what's next, at the core of what these conversations are is getting to understand the patient and getting to understand their level of knowledge. System 3, oncology nurse, frontline champion*<br><br>*"Asking the questions this way has...reframed for me....you can't actually say what the choices for treatment are if you don't actually know what is important to the patient, or what a patient is afraid of, or what they value the most." System 4, oncology physician frontline champion*<br><br>*"Distributing that emotional workload over the course of the patient's illness might be a way of decreasing that barrier to beginning that conversation. But it does mean that....it has to be part of your communication all along. I talk about it now as building the trust that a patient understands that you gather their goals and values continuously over the course of their relationship, that caring for their demented mother at home is the most important thing for them, and that receiving a chemotherapy that has even a small chance of getting them out of the hospital for a week or two is unacceptable in terms of what their long-term wishes are." System 3, oncology physician, clinical leader*	**Sub-themes:** Associations or conflation of serious illness communication as a discussion about dying, hospice, or life-sustaining treatment decisions; beliefs that that serious illness conversations have a pre-ordained outcome of withdrawing or limiting care; conflicted attitudes toward communication (e.g. concerns that decisions may conflict with the medical agenda to provide advanced therapies)<br><br>*"...when you mention the word...goals of care.. it's a deer in headlights for [hospitalists] often about- "We're going to do what? They're not dying." So there was really a lack of understanding about the benefits of having these early upstream conversations." System 2, inpatient palliative care social worker, frontline champion*<br><br>*A big thing as part of our project was instead of calling them goals of care or family meetings, we called them patient-centered care conferences....so that way, residents and the nursing staff didn't feel like ...'we're going to start withdrawing care.' System 3, oncology nurse, frontline champion*<br><br>*"We spent about a year planning for SICP... and were only a few weeks away from training the nurses when...one of the [surgeons]...said to me, "You can't ask these questions, because these patients may need a transplant and I don't want you to talk them out of a transplant." He felt that asking these questions would...undermine the goals of ... transplant... System 4, palliative care nurse practitioner, implementation team*<br><br>*This clinician associates serious illness conversations with hospice, and when that association is there there's less of a perspective of this is a conversation you can have to help a patient decide whether they want to go down the route of this particular treatment. System 4, oncology physician, frontline champion*<br><br>*We had circular conversations week after week about which patients to target...someone would say, "Well, what about fourth line chemo?...and then someone would say, "Well, actually, my patient isn't dying if they're on a fourth line of chemo." System 5, palliative care physician, implementation team*<br><br>*I think I've seen it come the farthest within our [primary care] nurse care management group. And part with that, is just their deep understanding in recognition of what a serious illness conversation is and what it entails and it's*

	"I have had many clinicians, from nurses, social workers and primary care physicians, reflect back that message without me prompting to hear it. 'This is not about code status. This is about learning what matters to people and then acting on it.' That is one big piece in which the way the cultures change. They have reframed their outcomes and now, the culture is driving in the right direction. The guide is not set to get a code status. The patient selection is not about choosing people who are about to die and need a code status conversation. The structure of the whole program is focused on teaching people that were actually working for something different here. System 2, palliative care physician, implementation team	*not. I think we are still up against this in the broader organization. People conflate code status and MOLST as a serious illness conversation when we're really talking more upstream. System 2, primary care program manager, implementation team*<br><br>*And I think that there is sometimes a difficulty in holding both...the drive for a cure and the limits that we face...Anything to question that, sometimes bumps up against the identity of being a discovery scientist. System 5, palliative care physician, clinical leader*<br><br>*And in a lot of places, surgeons, when a patient gets referred to a surgeon, they view themselves as the last hope for the patient for survival. So it's almost like most surgeons expect that the conversations of alternate treatment modalities has already occurred, and that they're there to be consented and to be scheduled for surgery. And I think that there has to be a paradigm shift in that thought process. System 1, surgeon, clinical leader*
Degree of empowerment to initiate conversations with patients	**Sub-domains:** Improved confidence and self-efficacy in initiating conversations with patients; enhanced acceptance, psychological safety, and trust for the role of advance practice clinicians, nurses, and social workers in serious illness communication; integration of values and goals discussions and goal-concordant care into role identity<br><br>*I think for clinicians who have not typically felt empowered to initiate the conversation, I think teaching them the very pragmatic parts of the conversation that will make the conversation acceptable, like setup, and delivering a prognosis, and then having a scripted list of questions, I think, has really helped clinicians who are previously very uncomfortable and didn't know where to start. System 4, palliative care and oncology physician, implementation team*<br><br>*I took the class and I did it after several of my colleagues had done the class and they had extremely wonderful feedback. They all came into it saying, "It's another meeting. What .. is this?" And they all came out saying, "Oh my god. This really worked."...They were pretty amazed about how much information they were able to get and glean just by doing those conversations. So I took the class. I was blown away because just in those 10 to 15 minutes I was like I could get all this information. System 1, hospitalist physician, clinical leader*	**Sub-domains:** Reluctance & nervousness about initiating conversations with patients; attitudes that serious illness communication is the physician's job, contributing to lack of psychological safety and trust for advanced practice clinicians, nurses and social workers to take an active role; feeling uncertain about roles in serious illness communication (e.g. sense of powerlessness)<br><br>*We had educational sessions where we did an abbreviated version of the Serious Illness Conversation training...A lot of the stuff that we got back from people was that they were afraid that this was working outside of their scope to start these conversations. ...And then also some people mentioned just their level of comfortability with the conversations and not having enough education to be able to actually do them. So I think being able to provide some of that education was very helpful. But even after we did our project, people identified that they wanted even more information, more education, System 3, oncology nurse, frontline champion*<br><br>*Then there's a department ... where the [leader] said, "No social worker may....ever have a conversation about end-of-life with any of our patients...." ....And I have rarely gone on to that [site]. System 2, palliative care social worker, frontline champion*<br><br>*And it was a group of....nurses... who would get the training... And so as we were scheduling those trainings, one of the [surgeons]...expressed that he felt it was not appropriate for nurses to be asking those questions of his patients...And so we*

	"....three of our physicians when they reflected on the things that have happened in the past two years in our practice that were the most culture-changing series, serious illness conversation was one of those things that was brought up. And they felt it's culture-changing because it empowered each person that was either involved in that conversation in any way but in particular physicians...to be able to have these really meaningful conversations with patients that they may have been nervous to have before just because they didn't have the tools to do it or like the appropriate language. And there was a culture change in just the way we approached it but also that there's a culture change in emphasizing our role in the palliative process - that it's not just like the palliative care team and us. It's all one continual spectrum that we play a really important role in that in our patients, particularly with advanced cancers. System 4, oncology physician, frontline champion<br><br>*Some of our younger physicians have taken this up like gangbusters. They have just exploded with this, with having up to 15 conversations per month....And talking to them about it, it seems that this has just given them the toolkit that they needed to be able to approach the conversations that they didn't know how to approach before. And I think giving them the structure and the phrasings was just a relief. So there's also a sense of relief that comes with the more routine ones, I find from my colleagues. I think what I've heard from those who really take it is, again, this relief at having a structure so they know where to go next and what to say next. And the phrasing is helpful so that they can have these easy phrases close at hand on the tip of their tongue and they're not groping for words and thinking more about, "What am I going to say next?" rather than focusing on what the patient is actually saying. And so that's the feedback that I've received from them. System 4, oncology physician assistant, frontline champion*<br><br>*"I never really understood that there were so many different ways to make a prognostic statement. That it's not necessarily a time-based statement all the time. It can be a functional statement, etc. That helps me a lot. One of the things that we talk about is that when [a patient] says they don't want to know how much time they have, a prognostic statement doesn't always mean that you have to give a time-based statement. So that has been a game changer for a lot of myself and my colleagues. System 4, oncology physician, frontline champion*	*decided – maybe now is not the time. System 4, palliative care nurse practitioner, implementation team*<br><br>*Some APPs feel empowered that they can have these conversations and are comfortable with their clinical knowledge to know what to say. Some do not feel empowered either because they don't feel like they have the personal knowledge or working with an attending where they feel like it's not appropriate, and the attending likes to have those conversations. System 4, oncology physician, frontline champion*<br><br>*Well, I think that the NPs and the social worker really felt that they were not supposed to document serious illness conversations, that it was much more the doctors. And so breaking down that barrier, wherever that came from. System 3 geriatrics project manager, implementation team*<br><br>*The reality is they were so enthusiastic to be trained and so willing to try it out. But actual use of that guide to have conversations changed very little, even with a lot of coaching and a lot of hands-on prompting and sort of revisiting and troubleshooting things. It came back to...that they didn't feel comfortable, they felt that having that conversation without the attendings' permission or presence or buy-in was overstepping their role in some way System 4, palliative care nurse practitioner implementation team*<br><br>*We trained nurse coordinators who work in the clinic, and again, they're also very engaged and excited. We trained a lot of [site] nurses, and they were asking me to put up the documentation poster in their work room and were thinking about ways to get other people engaged, and I followed up with that and didn't get any response from them via email, but they definitely called out verbally during the training that they don't feel comfortable doing this without a doctor asking them to, but they also said that they feel like this should be happening more. System 5, program coordinator, implementation team*<br><br>*Well, I think we kind of have a joke among social workers sometimes where we'll just say, "I don't know if certain positions value or respect our role here as clinicians, as part of a team, even." And so when you have that kind of culture at times, I already feel like we're a little bit at a disadvantage in terms of educating our colleagues. System 2, inpatient social worker, frontline champion*<br><br>*I have heard in some other groups that the APPs don't feel like, "Well, I can't have this conversation without the doctors really signing off," or, "That's not my*

	And then a lot of people complained about, "How am I going to get the time to do this in the day?" but then as it went on, and I'm including myself, I feel like the guide makes those conversations more efficient. A conversation that would have taken me longer before because I was trying to think about what questions to ask, now I don't have to think about them. What ended up happening was that people were like, "Oh, this is actually more efficient," and "Oh, I'm having a more thorough conversation." System 4, oncology physician, frontline champion<br><br>*I am noticing that it's tough to have these conversations with patients and having...a guideline for clinicians to hold onto gives them, I think, confidence. I think there's an increase of confidence with tackling these kind of conversations and an increase in comfort. And I also think there's an increase in just overall highlight on people's wishes and their goals and their boundaries of care, which I think has been very meaningful. System 1, palliative care nurse practitioner, frontline champion*<br><br>*"For us as nurses, we really weren't that confident that we were going to be kind of allowed to do this type of work. There was a lot of timidity in the work at the onset. Over time, it has become very integrated into the work we do. I don't wait to be directed to have the conversations. I take proactive steps. I will communicate the patient's goals to inpatient teams...I will seek out palliative care early on in the trajectory too. Your goal is to have concordant care with patient wishes so you have to speak up if you think people don't understand. System 2, primary care nurse, frontline champion*<br><br>*So mostly, I did feel safe but not in the beginning. It takes time to build that trust. I had the backing of two docs who trusted me almost unconditionally....There was just so much misunderstanding...and worry we're going to come in and say, "Your prognosis is horrible.' It did take a fair amount of time to just convince the hospitalists...what my goal was- defining my role...as the person who is working to make sure that the patient's treatment is aligned with their goals." System 2, inpatient palliative care social worker, frontline champion*<br><br>*In some places it works really well. So in [disease center], there's two doctors and two advanced practice providers and they're a very tight-knight, close group. They all sit in the same work room...they talk*	*place." So I think it varies by group how empowered the APPs feel and how safe they feel. And in the groups where they feel empowered and safe and that, then they have conversations and where they don't, then they don't.* System 5, *palliative care physician, implementation team*

	about their patients all the time. And in that group, when the clinical director was being supportive, they, the APPs, had the conversations and she fully trusted them to have the conversations, and they felt empowered to have the conversations. System 5, palliative care physician, implementation team<br><br>*I put in a self-referral and say, "Okay. I'm going to do the serious illness conversation with this patient," which everyone welcomes. The physicians are always like, "No problem. Go ahead. Go for it." System 2, inpatient social worker, frontline champion*<br><br>*I think that within the complex care team, there was no doubt that this became part of their standard work. So it's definitely different from where we started. I think in the beginning it was like, well, the oncologist has to talk to the patient, or the PCP hasn't had a conversation. Then within the interdisciplinary team, there was this feeling, I think, of empowerment but also an accountability of, "I know this patient. I talk to them as much or more than anybody. This is part of my role." So as part of that role identity, I think that's actually been a culture change even within the complex care team and I think for many of them that's a real empowerment. For some, I think it's still intimidating but it is part of their job description now. System 2, primary care physician, clinical leader*<br><br>*Early on, a lot of people were sitting around waiting for the doctor to do it and it wasn't getting done. We have seen over time and it is pretty clear from our data from the other day...about half of our conversations are done by physicians and about half by other professionals, which I think is critical. System 2, palliative care physician, implementation team*<br><br>*For me personally, I definitely think learning more about this has given me a sense of empowerment. I also take a lot of empowerment from just caring for patients. To me, being a patient advocate, as I mentioned, is my job...I need to speak up for what's right for my patients. And so that's for me, where I get a lot of empowerment. And I think that's where most nurses get their empowerment from, is knowing that they have the responsibility to do this for their patient.* System 3, oncology nurse, frontline champion	

	Think that some of the biggest successes are...beginning of decompressing the physician members of the team from being the exclusive and sole holders of the conversation. And I say beginning because I feel like we are still working on this extensively. I don't feel like it's the big, big, big win that we can have. But there's now much more agreement around ways to have others have conversations where physicians would-- they would be distressed around the amount of time they're spending, but then have qualms about other team members who aren't physicians having conversations about advance care planning. System 5, palliative care physician, clinical leader<br><br>*Within the... team, there was no doubt that this became part of their standard work. So it's definitely different from where we started....I think in the beginning it was like, well, the oncologist has to talk to the patient, or the PCP....Within the interdisciplinary team, there became this feeling of empowerment but also an accountability of, "I know this patient. I talk to them as much or more than anybody. This is part of my role." I think that's been a culture change...it is part of [nurse care manager] job description now. System 2, primary care physician, clinical leader*	
Perceived conversation impact	**Sub-domains:** Perception that earlier values and goals conversations lessens distress for patients; improvements in clinicians feeling effective in providing personalized care, supporting patient decision-making, and strengthened relationships and connection with patients<br><br>*I think the biggest successes come in the patient stories. We hear them a lot from our nurses specifically. And sometimes our patients or families. We hear about the anxiety relief that they're feeling that they've been able to have a conversation and how much more settled they may be feeling. And to me, It's hard to quantify, but those are the anecdotes that keep us going. System 2, primary care program manager, implementation team*<br><br>*"I've had plenty of these conversations where it hasn't been a crossroads. It's more been a check in... to make sure that we're on the same page....and the patients just express gratitude to be...able to express what their goals and wishes are. And I'm not concerned or worried that these words have never been said to the patient...System 4, oncology physician assistant, frontline champion*	**Sub-domains:** Concerns about taking away hope and raising patient anxiety or sadness; feeling overwhelmed by serious illness communication due to discomfort with surfacing and managing emotions and 'scarcity' in practice environments<br><br>*"Another factor is that, if the conversation appears out of the blue for a patient, and if we're asking them to have conversations in a non-crisis setting, a lot of our oncology physicians will say- "Why would we do it if there's nothing bad happening right now?" ...Are you going to signal to the patient that something bad is happening, even if it's not, and then is that anxiety going to somehow come back on them...basically creating anxiety that I now have to deal with."* System 4, palliative care and oncology physician, implementation team<br><br>*I think the thing that's missing from our oncology culture is acknowledging the contributions of either faculty wellness...and readiness to have these conversations....emotional energy is not an infinite resource...I still think that...it's a pain point to have these conversations...we probably need to give people more tools to respond to emotion in a way that feels authentic. Depending on where that [clinician] is starting from, I think that can really influence how much bandwidth*

	...one of the questions was, "How much information about what's likely to be ahead with your illness do you want to know?" and she said... "I don't actually want to know," but there were these teams coming in..and telling her prognosis. And the reason she was so anxious...was because she didn't want to talk about it...I could already see the impact because I went out, told the attending, told the resident...and that nurse saw the impact." System 2, inpatient social worker, frontline champion<br><br>I had a conversation yesterday with a patient's spouse. It's someone I've had multiple conversations with over the past couple of years, and it was helpful to review the different conversations I've had and what he had said, and to help her to see how he was changing his focus. That he was looking to be more at home System 2, primary care nurse, frontline champion<br><br>It's just been so well received by the patients and the family. I've had people I've had people tell me, "Thank you for considering this. Nobody has talked to us about what he or she wants. Nobody has talked to us about quality of life yet. And it's really made a great impact in our lives." And to me- that's why I went into medicine. System 1, surgeon, clinical leader<br><br>I operated on a gentleman that had a rectal cancer and recto-urethral fistula... The conversations that we had really centered around what he wanted long-term and what was his idea about the best quality of life that he could have. And it wasn't having a big operation....And what we decided to do was really focus on trying to control the growth of the tumor. And we did that for months...he's actually extremely happy with the results. System 1, surgeon, clinical leader<br><br>I think there's still moral distress. I think that's just the nature of this population [site] where providers in particular feel like this care is not going to help them in the way that the patient thinks it will. I think that's lessened a bit, though, because I think they're starting to hear more from the patient. If the patient's willing to go through all of these things then I feel a little bit more at ease knowing that they're willing to undergo this and they don't think that this is suffering, that they just feel like this is part of their care to get them to where they need to	*they have to have the conversation, because emotional energy is not an infinite resource. System 4, palliative care and oncology physician, implementation team*<br><br>We've also heard that, "I don't want to make my patient anxious. I don't want to make them cry. I'm nervous about saying this." Kind of revealing their own vulnerability. System 1, project manager, implementation team<br><br>Other huge barriers that have come up over and over having to do with clinician anxiety... [Primary care clinicians]...go from a... well person visit for a thirty-year-old to a serious illness conversation...I have heard from a number of them how jarring and...hard it is to personally prepare for that transition. System 2, palliative care physician, implementation team<br><br>I think the third factor, and I think we probably don't acknowledge this enough and maybe I need to have more empathy for this, but the actual pain involved for the clinician. So, these are deep and meaningful conversations, and I think they do cause some sadness for some patients, or at least make them think about difficult topics, and I think oncology clinicians don't want to introduce more pain, because they're already keenly aware that so many of our patients are suffering all the time, even when they're, quote, doing well. So I think the sort of emotional pain involved is a barrier. System 4, palliative care and oncology physician, implementation team<br><br>And just a lot of discomfort around the emotions around it. Like I don't want to make them sad. I don't want to make them upset. I don't want families to be upset. So I think a lot of discomfort came out in that way of like why do we have to push beyond the envelope. It very much felt like this is not within our scope and that other people before us had this conversation. Our job is really just to get them through this day and they go off to rehab or they go home or wherever. System 4, palliative care and geriatrics physician, implementation team<br><br>And so for all of us doctors who are afraid of emotions, when somebody plops it in your lap, usually we're pretty good about taking it. But it's also easy to sort of tiptoe around it if we have 15 minutes and a lot of other things to talk about. System 3, hospitalist physician, implementation team<br><br>I've heard this from [disease group], from -- and a few others -- is just it's emotionally draining. And they feel like they will only have these conversations with patients they have like a personal rapport with, and that they will not

	be. So I think the stress has lessened slightly. System 4, palliative care and geriatrics physician, implementation team<br><br>*So I think it reset his expectations and his bias or opinions...It was really eye-opening for him to be like, 'Oh, the patient actually understands what's going on and is totally okay with it. So if the patient's okay with it, I should maybe be okay with it, too. Because it's really the patient's decision.' So I think it helped him just be at peace with the patient's decisions a little bit more. System 4, palliative care and geriatrics physician, implementation team*<br><br>*So I think allowing that SICP conversation to happen allowed [the family] to tell a story to personalize [the patient], and it makes it a lot easier for them to guide decision-making realizing that you can help him by potentially supporting him through this difficult time and keeping him comfortable. System 1, hospitalist physician, frontline champion*<br><br>*One of the PCPs...was talking about a patient that that...he was triggered to have one of these conversations and just came out reflecting on his own feelings...He had not really seen that they were getting sicker and that he felt he knew them so much better and that had learned things about the person that he didn't know....he clearly was activated by the encounter that he had with this person, it deepened the relationship that he had with him and I have heard from a lot of the primary care physicians that they go into primary care because they enjoy those kinds of relationships...the positive feedback...reconnects them somewhere within themselves with some of the reasons they really love the work. System 2, palliative care physician, implementation team*<br><br>*Doing the serious illness conversation every day, it becomes a part of who you are as a practitioner, as a person. And I think that is what the meaning is to my practice now is having these conversations early and establishing truly trusting relationships. I've had patients that I knew for seven, eight years but I never really knew them. I knew their medical stuff really, really good. But I never really knew their fears and their hopes and what is the boundaries. System 1, inpatient palliative care nurse practitioner, frontline champion*	*otherwise have the conversation. And then she ....said that there was a patient who she knew for many, many years, and having a conversation with him was so emotional and so draining. I think she felt like there was a personal connection there, that she felt more impacted by having the conversation. And she said, at the end it was good that they had the conversation, but it was just a lot of tears, basically. System 5, program manager, implementation team*<br><br>*Number one I think there is an emotional discomfort. Nobody actually likes to give that information and nobody likes to talk about these things. And so I think a coping mechanism for providers is to avoid it as much as possible...I think number one is avoidance because of emotional discomfort. System 4, oncology physician assistant, frontline champion*<br><br>*I think the biggest challenges are...the current structure of... reimbursement and time in the ambulatory setting....And this sense of "It's such a big thing to do..."...it's overwhelming....people don't have the luxury of a 30-minute appointment or sometimes even 20. System 5, palliative care physician, clinical leader*<br><br>*I think for a lot of people who feel like, "This is the right thing to do. There's just not an extra minute in my day or in my schedule to do this" System 2, primary care physician, clinical leader*<br><br>*I have proposed this to our cancer center leadership, and this has not gained traction...that all clinicians...should have 30 and 60 minutes slots allotted for returns and news...I think that we need to change the culture around how we're spending time and how many patients were expected to see. System 4, palliative care and oncology physician, implementation team*<br><br>*The probability of me having a SICP conversation on a Saturday when I have 24 patients is 0, but if you gave me 15 and it's a Wednesday, I can guarantee you would be the easiest thing to sit down and make the time for it. System 1, hospitalist physician, clinical leader*<br><br>*The other part of it is that these conversations are hard to have. And...among everything else that we do, it has to be felt to be a core element of care and not a burden that is an additional ask of an already burdened clinical population. System 3, oncology physician, clinical leader*

	"This is what we should be doing as social workers. It's bringing us back to our roots that we're not just here to hand out substance use resources.." System 2, inpatient social worker, frontline champion<br><br>*It's one of those patients that will stick with me forever, and I came in and he wasn't doing very well....And I went into the room and I had a conversation with his wife...I ended up paging the attending and this is the attending that ...[had] a hard time with these conversations with patients, and I said to him, "Please talk to me before you go into the room." And so I talked to him and I said, "This is what's going on. This is where the wife is at. She really needs somebody to tell her it's okay to let him go." That for me, was such an incredible experience because he listened, he saw what was going on in the room, and he agreed with me. And it was just that moment that-- I think even after working with him after that point, I think he had a new level of respect for me, and I had another level of respect for him...It was just incredible to be able to work with him and for us to be able to do what was best for that patient. System 3, oncology nurse, frontline champion*	*[a colleague]...emailed us a couple days ago and said, "I'm so sorry...we've just been so busy...I want to do this. I know it's important. I can't even get through my clinic day. System* 5, palliative care physician, implementation team<br><br>*"I think everyone just feels too crushed right now. There's too many quality metrics, You have to do distress screening; you have to stage...now it feels like there's 20 other things that are being asked. Yesterday I had a conversation with [colleague], just to give you an example...and he's like "I'm going to tell you right now, if you ask me to do one more thing, the answer is no." And I was like, "Okay." System 5, oncology and palliative care physician, clinical leader*<br><br>"*We have to take care of so many things from blood pressure management to diabetes to coronary artery disease to skin rashes and depression. And okay, how many minutes do we have left to talk about [serious illness communication]? System 3, geriatrics physician, frontline champion*
Practice norms	**Sub-domains:** Earlier and longitudinal serious illness conversations; Enhanced focus on person-centered domains, including patients' values, goals, hopes, and worries; reliable processes and shared responsibility and accountability to integrate serious illness communication into routine care<br><br>*And so I think the biggest change I've seen is that it's been much more of a proactive approach...with the patient being involved versus families having to make the decision when the patients too sick to participate. System 1, hospital physician, clinical leader*<br><br>*In the beginning, more of them were happening in the hospital where the patients were transitioning to hospice and going home. They were really at the end of life at that point. And over time...you recognize when these conversations should and need to happen. Conversations are certainly happening a lot earlier in the trajectory so there is a greater understanding of what the patient wants as you get into more difficult times. System 2, primary care nurse, frontline champion*<br><br>*The conversations about goals are richer, both with me and with the patients and families. The way they present it is no longer, "Well, we really need you to help with code status and goals of care." It's more*	**Sub-domains:** Avoiding conversations until a crisis or poor prognosis at the end of life; content of the discussions focused on medical agenda and acute decisions, e.g. hospice, code status, life-sustaining treatment preferences; lack of reliable team processes and lack of clarity about team roles (sense of 'chaos')<br><br>*Providers avoid it as much as possible until it becomes so blatantly obvious they can't avoid it anymore or...until... the patient gets hospitalized or goes to the E.R. with really bad complications. System 4, oncology physician assistant, frontline champion*<br><br>*"In the past, serious conversations were usually a reactive process versus proactive. Oftentimes people came into the hospital sick with the expectation of getting better, and when things didn't, there was a reactive process...about making critical decisions when patients are heading down the tubes quickly. System 1, hospitalist physician, frontline champion*<br><br>*In the beginning, more of them were happening in the hospital where the patients were transitioning to hospice and going home. They were really at the end of life at that point. System 2, primary care nurse, frontline champion*<br><br>*I think there was a basically general perception - probably is the best word - of conversations regarding advance care planning and goals of care just not*

	like, "Well, I had this conversation with the patient, and it sounds like he's struggling with X, Y, and Z. And he really has this goal, but he's also worried about...System 4, palliative care nurse practitioner, implementation team<br><br>*Content of the discussions that we're having with patients and moving those from, honestly, I think being focused very much on life-sustaining treatment preferences and code status to more values-based discussions about goals, preferences, patient values, is one of the things that I think about in terms of concrete changes, or a culture change and getting providers to be less hung up on thinking about code status, and more about kind of bigger picture goals and values. And I think we see that in the documentation that we're collecting both in terms of the uptake of SICP notes in the health system. And then also just kind of other advanced care planning notes. I think that they're happening earlier, and there are more conversations per patient....you can see the goals changing. And I think ...another change is moving these conversations from the inpatient setting, and the ICU, specifically to the outpatient clinic. System 4, palliative care physician, implementation team*<br><br>*I see that being used a lot by my other colleagues now. It's like, "What are some of your fears?" ...So confidently. And I think that pattern of communication has really changed so much in my hospital. "What are your fears? What gives you strength?" System1, palliative care nurse practitioner, frontline champion*<br><br>*Because I went back and looked at the data, and [hospitalist] just took off. And it's like for hospitalists, it's probably a little bit harder to have these conversations because you do not have a longitudinal relationship with your patients like in primary care or oncology. Because you're seeing them for a very short amount of time. So he was able to really get through all of the hurdles that most people will say like, "I don't know them. I don't feel comfortable talking to them about this." But he found a way to normalize it and make it part of his practice. System 3, hospitalist physician, implementation team*<br><br>*When you enroll your patients, you do ask your physicians the surprise question, which triggers that kind of thought process that, okay, we have work to do. Me and you have work to do. If you're telling me you*	*happening for a multitude of reasons. And I think the loudest voices around the distress of this were in that inpatient side of things for clinicians encountering patients who would be bewildered when they became very sick with an uncertainty around their future and were caught where-- I think the clinicians in particular felt a lot of the stress around being the first to have these conversations with patients that they didn't know. And also feeling like they couldn't change much for these patients with regards to their care because conversations were happening so late. System 5, palliative care physician, clinical leader*<br><br>*We had the gap of not assessing understanding. We had the gap of not truly sharing prognosis. And then we had the gap of not obtaining information from the family or sometimes from the patient about their values. System 1, palliative care physician, implementation team*<br><br>*We have these poor outcomes after surgery or the patient might fail. And the family was-- they really thought that surgery was going to fix them and it didn't, so now you're going to tell me that my loved one is dying. And then that would create a lot of tension and stress between family members and the medical team. And then they would call for palliative care to fix it. "Go talk to them. I don't know why they're not understanding." And we're like, "Did [you]... explain to them how sick they were?" And, well, they were really sick and it was a bit obvious. I know, but did you communicate that to them? Did you tell them, "This is what it is? And these are your potential outcomes," with or without surgery? And so how did you go about that?...And it was creating a lot of anxiety between nursing staff members. System 1, palliative care nurse practitioner, frontline champion*

	wouldn't be surprised if this patient died in two years, then we need to have that conversation. And I make that clear...I say, "Okay, you say you wouldn't be surprised. That means we have to have this conversation." So it becomes kind of a team effort early on...It forces you to-- you got to tackle it. You can't ignore it. I don't want to do it. It might be really hard but it just kind of keeps you on track that people still need this type of conversation and I just feel that it's our duty. It's our duty to do it. System 2, primary care nurse, frontline champion<br><br>*They don't know how to hold these conversations, so they consult us for these conversations because they know we help in care planning and palliative care. And they bring us in....And so for a particular group...of interventional cardiologists, we are on board for every single one of their high-risk PTIs, they will not do surgery without us first doing our evaluation and our evaluation incorporates SICP. System 1, palliative care nurse practitioner, frontline champion*<br><br>*Early on, this would have been the doctor's job and a lot of people were sitting around waiting for the doctor to do it and it was not getting done. Now, they are all in there and they had permission to be in there. They understand and I have heard lots of stories about how it is so helpful. We have seen over time and it is pretty clear at our data from the other day. About half of our conversations are done by physicians and about half by other professionals, which I think is critical. System 2, palliative care physician, implementation team*<br><br>*We have three or four APPs ...they share their patient panels with us and they saw somebody that they thought would need a conversation. They would be like, "Hey, I see that you have restaging scans coming up." We built in different scenarios in a template like a smart phrase, and they would say, "Hey, I think you have this coming up. This is a really good time...to have a conversation with Dr. So and So about what's important to you and your cancer care." ...and that has also increased the number of conversations we've had. System 4, oncology physician, frontline champion*<br><br>*I think within the hospitalist group in particular, they were really good about—'if I didn't get the conversation done I'm going to put that in my sign out because I expect the next person to get that done.' So this is a shared responsibility and ideally the person admitting the patient*	

	will do it but if they can't the next person really needs to do it....I think they felt very much an ownership of this and held each other accountable for like, "Hey, did you see that the new patient didn't have a conversation? ... I can cover the next two admissions so you can get that stuff done...." We have almost 100% uptake of the guide for documented conversations for patients admitted to the [site]. So every patient that gets admitted, gets a conversation. System 4, palliative care and geriatrics physician, implementation team	
